# Providing the Best Infectious Diseases and Primary Care to Our Patients With HIV: Time for Delivery Models to Evolve

**DOI:** 10.1093/cid/ciag096

**Published:** 2026-03-27

**Authors:** Christopher J Graber, Raphael J Landovitz, Westyn Branch-Elliman

**Affiliations:** Infectious Diseases Section, VA Greater Los Angeles Healthcare System, Los Angeles, California, USA; Division of Infectious Diseases, David Geffen School of Medicine at the University of California, Los Angeles, Los Angeles, California, USA; Center for the Study of Healthcare Innovation, Implementation, and Policy (CSHIIP), VA Greater Los Angeles Healthcare System, Los Angeles, California, USA; Center for the Study of Healthcare Innovation, Implementation, and Policy (CSHIIP), VA Greater Los Angeles Healthcare System, Los Angeles, California, USA; UCLA Center for Clinical AIDS Research & Education, David Geffen School of Medicine at the University of California, Los Angeles, Los Angeles, California, USA; Infectious Diseases Section, VA Greater Los Angeles Healthcare System, Los Angeles, California, USA; Division of Infectious Diseases, David Geffen School of Medicine at the University of California, Los Angeles, Los Angeles, California, USA; Center for the Study of Healthcare Innovation, Implementation, and Policy (CSHIIP), VA Greater Los Angeles Healthcare System, Los Angeles, California, USA

**Keywords:** HIV, primary care, healthcare delivery, specialty care, health policy

## Abstract

Advances in antiretroviral therapy have substantially increased life expectancy among people with human immunodeficiency virus in the United States, leading to an older population with a growing burden of chronic noninfectious comorbid conditions. In parallel, the delivery of primary care to an older population is increasingly complex, often taking infectious diseases (ID) specialists outside their areas of expertise. These concurrent trends highlight the importance of integrated care approaches. Comanagement between ID specialists and dedicated primary care providers offers a framework to optimize comprehensive care for this population.

## Teaser

Advances in care for patients with human immunodeficiency virus have simplified the management of infectious disease–related conditions, yet primary care has grown more complex. Integrated care models that turn over nonspecialized care to primary care providers are needed.

One of the things that makes the field of infectious diseases (ID) so exciting despite all of the challenges facing the specialty is that it is always changing—new pathogens emerge, new treatments are introduced to the market, innovative paradigms and diagnostics develop—and ID providers are at the front line of forever learning and adaptation. In most cases, ID steps in, leads until the situation improves, and then ID steps back and steadies itself for the next big threat. Which brings us to the human immunodeficiency virus (HIV) epidemic.

In June 1981, the Centers for Disease Control and Prevention published what is now recognized as the first reports of HIV in the United States among a cohort of patients in Los Angeles [1]. The scope and severity of the HIV epidemic soon became clear: from 1992 to 1995, it was the leading cause of death among men 25–44 years old [2]. In the 1990s, people with HIV (PWH) were typically young, and their most common presenting condition was an opportunistic infection or cancer.

In 1996, so-called “highly active antiretroviral therapy” was demonstrated to suppress HIV replication and allow persons with extreme immunocompromise to undergo immune reconstitution. This revolution has been followed by the breakthrough that viral suppression unequivocally prevents transmission to sexual partners, strategies for preexposure prophylaxis, and the relatively recent introduction of long-acting antivirals. Each milestone serves as a testament to the power of biomedical research, strong public health, and good public policy to improve people's lives.

In parallel with these advancements, the population of PWH has also changed. Their modal age in the United States is 55–64 years [3]. Many HIV trials now focus on *noninfectious* issues, such as the role of statins as primary prevention, frailty, and metabolic syndromes [4]. With the antiretroviral regimens now on the market, the life expectancy of PWH continues to improve—nearly matching that of people who do not have HIV if PWH are appropriately treated and managed absent profoundly low CD4 cell counts—but with the caveat that PWH develop medical comorbid conditions about 10 years earlier than risk-matched HIV-negative individuals [5, 6].

Change can be difficult to recognize and even harder to act upon, particularly when it occurs gradually. For years, antiretroviral therapy management demanded highly specialized expertise: clinicians needed to balance virologic potency with toxicity, drug-drug interactions, comorbid illnesses, complex resistance patterns, and opportunistic infections. This complexity made comprehensive HIV care—including primary care—most naturally and appropriately the domain of ID specialists.

Since those care delivery models were established, however, antiretroviral regimens have become more potent, more convenient, and safer. The vast majority of PWH in care have achieved and maintain virologic suppression with a 1-pill-daily regimen. HIV management is now more straightforward for both clinicians and the PWH whom we serve. As survival has improved, patients now live long enough to face the full spectrum of age-associated conditions. This progress has brought with it new challenges: the need to address routine cancer screening, bone health, and increasingly common chronic comorbid conditions, such as diabetes, heart failure, hypertension, arthritis, and chronic obstructive pulmonary disease. These conditions have themselves grown more complex to manage. Diabetes, for example, now has >2 dozen approved glucose-lowering agents and numerous insulin formulations; this is not a disease for which most ID specialists have remained up to date on the optimal management. In addition, primary care practices may include robust support staff and systems to enable management of non–ID-related problems, including the need to meet quality metrics for diabetes and hypertension control, along with cancer screening. Simple ID-related metrics, such as the proportion of patients receiving recommended vaccines, are only a small part of a comprehensive primary care quality package.

**Figure 1.**
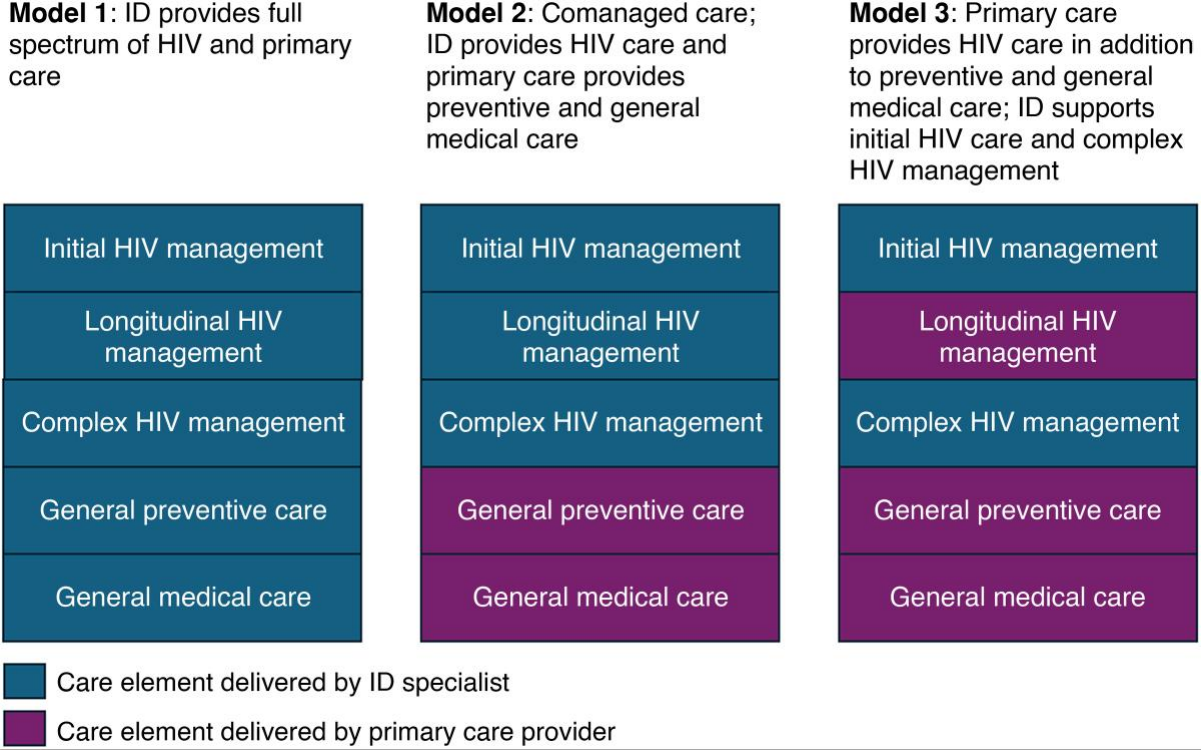
Clinical care delivery models for persons with human immunodeficiency virus (HIV) according to provider type. Abbreviation: ID, infectious disease.

These advances in HIV therapy, with a shift of routine HIV care from managing antiretroviral therapy and opportunistic infections to the chronic diseases of aging, raise important questions about the optimal model for providing both HIV longitudinal management and primary care services to this population in 2025. Three idealized general clinical care models can be considered (Figure 1).

Model 1 is a continuation of the long-term status quo: ID physicians continue to provide both ID care and all primary care services for PWH. Under this model, it is incumbent upon ID providers to put in the time and effort to keep abreast of latest advances in the care of common comorbid conditions, develop systems for tracking and monitoring recommended screening, and generally maintain general internal medicine expertise. Providers need to recognize that skills that are not used tend to rust and that if primary care is not a primary focus of our day-to-day work, we risk offering our patients lower-quality primary care services than they would receive from practitioners who focus in this area day in and day out.

Model 2 involves comanagement between ID physicians and primary care providers. Under this model, ID physicians are responsible for longitudinal HIV care and infectious comorbid conditions, such as sexually transmitted infection, with oversight of PWH-specific preventive care needs, such as specialized screening and vaccination recommendations. Management of noninfectious conditions, such as diabetes, and general preventive care, such as colon cancer screening, is provided by primary care practitioners. Model 2 likely represents care delivery systems for most chronic conditions of at least modest complexity that require subspecialty care in the United States, where specialists provide long-term management and follow-up for conditions within their specialty's focus but do not manage other aspects of care. For example, oncologists manage cancer treatment and long-term cancer-specific follow-up care but generally do not manage other chronic conditions, such as hypertension.

Model 3 treats uncomplicated HIV infection as a straightforward medical condition that can be managed by primary care providers, similar to other common medical problems with well-delineated treatment options. Under this model, ID physicians are consulted early in the disease course but once a patient has achieved stable viral suppression (with a simple regimen), management of both general medical care and HIV care is transferred to primary care, with ID involvement only continuing in medically-challenging cases. This model treats HIV as similar to conditions like hypertension, diabetes, and thyroid disease: specialists get involved only if the disease is complicated, and once patients with a complex condition are stabilized, their care is transitioned back to primary care. For example, an endocrinologist may be consulted to assist with initial titration and control of diabetes medications, but once a patient's glucose management has stabilized, ongoing care is often provided by a primary care physician. This model may become more common, particularly in areas with limited access to ID-trained physicians. In some regions of the United States, non–ID-trained HIV specialists are common and may also be able to play primary and/or consultative roles in this model.

Identifying the optimal HIV care delivery model for the current era means examining the current state of the field of ID and the broader context of the US healthcare system. ID is one of the smallest specialties in all of internal medicine [7]. Recruitment is challenging [8]. The ID workforce and ID knowledge are limited resources, raising the question of how this limited resource is best deployed. ID providers need to stay up to date on the ever-evolving field of ID and focus our expertise in areas where we will have the biggest impact. Antibiotic-resistant infections are associated with >4 million deaths per year worldwide [9]. Healthcare-associated infections, if they were counted as such, would be a leading causes of death in the United States [10].

The myriad ways in which the immune system can be iatrogenically compromised to serve various purposes (transplantation, autoimmune disease) have substantially increased complex infection management needs and expanded the need for ID expertise in transplantation and immunocompromised populations, another population of patients that will require long-term ID follow-up and engagement [11, 12]. In pediatric infectious diseases especially, vaccine-preventable diseases like measles are making a comeback, and recent changes to the recommended vaccine schedule will complicate early management and prevention decisions [13–15]. ID physicians are now increasingly needed to provide guidance and clarity about topics that were previously supported by other sources of expertise. Keeping up with all of these different aspects of ID inherently leaves less room for staying up to date with the rapid advances in other specialties and maintaining high-quality primary care clinical skills.

Recruitment into the ID field is also not keeping up with the ongoing demand for comprehensive care for PWH. This, coupled with the retirement of the original generation of HIV/ID specialists, is causing a workforce shortage in HIV care [16]. These changes lead us to call for a shift from the more historical model 1 to model 2 or, increasingly, a model 3 approach wherein the expertise of both generalists and ID specialists is used to its highest potential. We realize that primary care in the United States is in a state of crisis and that we must carefully work with our colleagues to design and implement care delivery models that are compatible with the realities of current health systems [17]. We also recognize that many PWH have intersecting social problems involving mental health, substance use, and inadequate housing that are difficult to manage yet are familiar to us in ID, so a single care model may not necessarily fit all patients and clinical care environments.

So, what should we in ID do with our time if we are no longer furrowing our brows as to whether we should prescribe a dipeptidyl peptidase 4 inhibitor versus a sodium-glucose cotransporter 2 inhibitor, doing our quarterly internal medicine maintenance of certification modules, or renewing tamsulosin prescriptions? ID has many ways to expand its influence and impact, and we can look to other specialties for how to adapt.

No clinician not trained in oncology would ever dream of prescribing chemotherapy, and no clinician not trained in rheumatology would reach for the complex immune suppression needed for a patient with lupus and acute kidney failure. We need to outline conditions and treatments that are clearly managed and “owned” by ID. Stewardship is just one example where we can take a more proactive ownership role to improve quality of care at the patient level and protect population health. Ironically, while the ID field largely still owns the domain of antiretroviral management (especially in cases with resistance mutations or toxicity), we still allow any provider to order and interpret cultures and prescribe most antibiotics—even though, in contrast to HIV management, antimicrobial management gets *more* challenging as antimicrobial resistance continues to worsen. While recognizing that it is impractical for ID practitioners to oversee every antibiotic prescription in the inpatient or outpatient setting, we need to explore exclusively prescribing (not just approving) most agents that are typically reserved for multidrug-resistant infections. Development of enforced criteria for use of most other antimicrobial agents should be well within our purview. Similarly, with regard to diagnostic stewardship, all healthcare systems should have dedicated salary support for ID practitioners to work with informatics teams to ensure proper deployment of infectious disease–related diagnostic tools, including newer, more expensive molecular tests such as microbial cell-free DNA testing.

Shifting to new care models focused more specifically on ID care rather than general medical care will work only if we simultaneously identify additional avenues for revenue and reimbursement. Again, other specialties hint at potential models and strategies for ID providers. Cardiologists can receive approximately 0.17 wRVU (work relative value unit) for electrocardiographic reviews—why should ID be any different with any blood, urine, or sputum culture that is positive? We need to be creative about identifying ways to support our efforts in healthcare epidemiology, public health, communications, transplant ID, and the other areas where we are contributing our knowledge and expertise, and we also need to do a better job of advocating for the value of these activities.

As we move into a world most recently shaped by the COVID-19 pandemic and sure to be shaped by epidemics as yet unidentified, ID practitioners need to take stock of where our field has been (particularly since the discovery of the HIV pandemic 44 years ago), where it is now, and where it needs to go. We should celebrate the success of nearly 30 years of highly effective antiretroviral therapy and recognize that times have changed. We have entered an era in which PWH are typically middle-aged or elderly with high cholesterol, high blood pressure, diabetes, and/or cancer; ID specialists are well suited to handle the HIV but not the other conditions. The time has come to pass primary care services for PWH to our primary care colleagues. Effective transitions to these types of models will require substantial coordination with our colleagues in primary care and consideration of how to address access challenges external to the field of ID but still affecting our patients. Regardless, long-lasting connections cultivated with our patients can still continue, and we can still rely on our expertise and experience in helping PWH live full lives. This balanced approach gives our patients high-quality ID care and high-quality primary care and also gives us time to focus on new challenges lurking on the horizon—a rare win-win in medicine.

## References

[1] Centers for Disease Control . Pneumocystis pneumonia—Los Angeles. MMWR Morb Mortal Wkly Rep 1981;30:250–2. 6265753

[2] Centers for Disease Control and Prevention . Update: mortality attributable to HIV infection among persons aged 25-44 years—United States, 1994. MMWR Morb Mortal Wkly Rep 1996; 45:121–5. 8622619

[3] US Centers for Disease Control and Prevention . HIV diagnoses, deaths, and prevalence: 2025 update. Available at: https://www.cdc.gov/hiv-data/nhss/hiv-diagnoses-deaths-and-prevalence-2025.html. Accessed 8 October 2025.

[4] Sax PE . REPRIEVE trial highlights shift in HIV care from ID to general medicine: observations in HIV and ID. **2023**. Available at: https://blogs.jwatch.org/hiv-id-observations/index.php/reprieve-trial-highlights-shift-in-hiv-care-from-id-to-general-medicine/2023/07/26/. Accessed 17 December 2025.

[5] Collins LF, Armstrong WS. What it means to age with HIV infection: years gained are not comorbidity free. JAMA Netw Open 2020; 3:e208023. 32539147 10.1001/jamanetworkopen.2020.8023

[6] Marcus JL, Leyden WA, Alexeeff SE, et al Comparison of overall and comorbidity-free life expectancy between insured adults with and without HIV infection, 2000-2016. JAMA Netw Open 2020; 3:e207954. 32539152 10.1001/jamanetworkopen.2020.7954 PMC7296391

[7] American Association of Medical Colleges . U.S. Physician Workforce Data Dashboard. Total physicians by specialty: United States and its territories, 2023. Available at: https://www.aamc.org/data-reports/report/us-physician-workforce-data-dashboard. Accessed 8 October 2025.

[8] The Match . Results and data: specialties matching service, 2025 appointment year. Available at: https://www.nrmp.org/match-data/2025/02/results-and-data-specialties-matching-service-2025-appointment-year/. Accessed 8 October 2025.

[9] Naghavi M, Vollset SE, Ikuta KS, et al Global burden of bacterial antimicrobial resistance 1990–2021: a systematic analysis with forecasts to 2050. Lancet 2024; 404:1199–226. 39299261 10.1016/S0140-6736(24)01867-1 PMC11718157

[10] Klevens RM, Edwards JR, Richards CL Jr, et al Estimating health care-associated infections and deaths in U.S. hospitals, 2002. Public Health Rep 2007; 122:160–6. 17357358 10.1177/003335490712200205 PMC1820440

[11] Black CK, Termanini KM, Aguirre O, Hawksworth JS, Sosin M. Solid organ transplantation in the 21st century. Ann Transl Med 2018; 6:409. 30498736 10.21037/atm.2018.09.68 PMC6230860

[12] Martinson ML, Lapham J. Prevalence of immunosuppression among US adults. JAMA 2024; 331:880–2. 38358771 10.1001/jama.2023.28019 PMC10870224

[13] WWNO New Orleans Public Radio . Louisiana's deadly whooping cough outbreak is now its worst in 35 years. **2025**. Available at: https://www.wwno.org/public-health/2025-09-03/louisianas-deadly-whooping-cough-outbreak-is-now-its-worst-in-35-years. Accessed 8 October 2025.

[14] Mississippi State Department of Health. Pediatric pertussis death reported. **2025**. Available at: https://msdh.ms.gov/page/23,30402,341.html. Accessed 29 September 2025.

[15] US Department of Health and Human Services. ACIP recommends individual-based decision making for hepatitis B vaccine for infants born to women who test negative for the virus. **2025**. Available at: https://www.hhs.gov/press-room/acip-recommends-individual-based-decision-making-hepatitis-b-vaccine-birth-dose-infants-born-women-test-negative-virus.html. Accessed 17 December 2025.

[16] Norberg A, Nelson J, Lin H, et al A forecast of the HIV clinician workforce need in the United States: results of a quantitative national survey. J Assoc Nurses AIDS Care 2024; 35:486–94. 39480051 10.1097/JNC.0000000000000495

[17] Rosenbaum L . Is a long-simmering crisis boiling over? U.S. primary care today. N Engl J Med 2025; 393:1537–41. 41099398 10.1056/NEJMms2510425

